# Anticoagulants is a risk factor for spontaneous rupture and hemorrhage of gallbladder: a case report and literature review

**DOI:** 10.1186/s12893-018-0464-6

**Published:** 2019-01-05

**Authors:** Zhilong Ma, Bin Xu, Long Wang, Yukan Mao, Bo Zhou, Zhenshun Song, Tingsong Yang

**Affiliations:** Department of General Surgery, Shanghai Tenth People’s Hospital, Tongji University School of Medicine, Shanghai, 200072 China

**Keywords:** Anticoagulants, Gallbladder, Spontaneous rupture

## Abstract

**Background:**

The spontaneous rupture of the gallbladder is extremely rare, majority of ruptures occur secondary to traumatic injuries. Here, we report a case of spontaneous rupture of the gallbladder with probably cause of oral anticoagulants.

**Case presentation:**

A 51-year-old woman presented to the emergency room with sudden-onset severe abdominal pain, as well as hypotension and low level of hemoglobin. Abdominal computed tomography (CT) scan showed a 2.5 cm filling defect and discontinuity in the wall of the gallbladder body, and a massive hematocele in the abdominal cavity. Past medical history was significant for hypertension and had been taking daily aspirin for the past three years because of interventional surgery for cerebral aneurysms, but no history of recent abdominal trauma or past episodes of biliary colic. The patient underwent an urgent laparoscopic abdominal exploration and the gallbladder was removed. The pathology just showed chronic cholecystitis and the patient recovered well.

**Conclusion:**

Long-term use of anticoagulants may increase the risk of gallbladder rupture and hemorrhage, which is a lethal condition. Rapid diagnosis and timely surgical intervention are the most important measures to treat the patient.

## Background

The spontaneous rupture of gallbladder is extremely rare, the majority of cases have followed penetrating war injuries and trauma [1, 2]. Rupture and hemorrhage of gallbladder is a lethal condition, rapid diagnosis and treatment are very virtual. Computed tomography (CT) scans and intravenous contrast association a careful medical history and physical examination frequently could help make an accurately diagnose. With the progress of medicine, the past exploratory laparotomy has been made a secondary consideration, and laparoscopic exploration is widely adopted. Here, we describe the case of gallbladder rupture in a patient with cholelithiasis who has been on anticoagulation therapy for two years.

## Case presentation

**Fig. 1:**
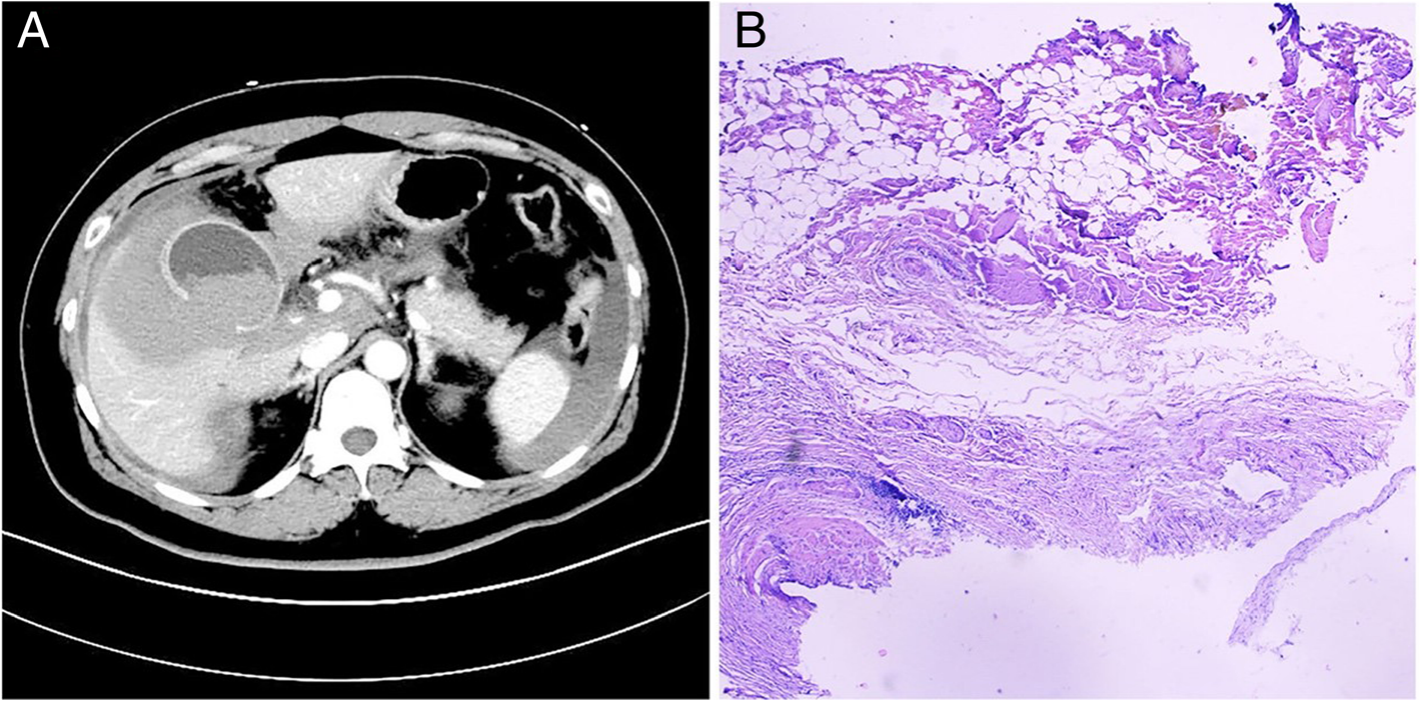
**a**, Abdominal CT scan with intravenous contrast showed a 2.5 cm filling defect in the body of gallbladder wall, a 1.0 × 0.8 cm stone in the neck of gallbladder and a massive hematocele in the abdominal cavity. **b**, Final pathology showed a disruption in the muscularis propria of partial gallbladder wall and plenty of eosinophils and lymphocytes infiltration in the mucous layer, associated with chronic cholecystitis

A 51-year-old woman presented to the emergency department with sudden-onset severe abdominal pain, as well as hypotension (75/48 mmHg) and diffuse abdominal tenderness with guarding on physical examination. Laboratory tests were significant for downtrending hemoglobin levels (75 g/L). Abdominal computed tomography (CT) scan with intravenous contrast showed a 2.5 cm filling defect and discontinuity in the wall of the gallbladder body, a 1.0 × 0.8 cm stone in the neck of the gallbladder, and a massive hematocele in the abdominal cavity (Fig. 1a). Past medical history was significant for hypertension but no history of recent abdominal trauma or past episodes of biliary colic; social history was not significant for any alcohol or tobacco use. Patient had also been taking daily aspirin (200 mg per day) for the past three years because of interventional surgery for cerebral aneurysms. The patient underwent an urgent laparoscopic abdominal exploration. A 2.0 cm defect was identified in the body of the gallbladder and an active arterial bleeding site was visualized at the edge of the defect. The remainder of the gallbladder wall appeared normal without any hyperaemia and edema. 2500 mL of fresh and clotted blood mixed with bile was evacuated from the gallbladder fossa, right supra-hepatic space, splenic recess and pelvic cavity. Final pathology demonstrated a disruption in the muscularis propria of a portion of the gallbladder wall and the abundance of eosinophils and lymphocytes infiltration in the mucosal layer, associated with chronic cholecystitis (Fig. 1b). The patient was discharged on post-operative day 7 without complications and recovered well.

## Discussion and conclusions

The spontaneous rupture of the gallbladder is extremely rare. While majority of ruptures occur secondary to traumatic injuries, the incidence remains low, at less than 2%, after abdominal trauma [1, 2]. Spontaneous rupture of the gallbladder can occur by the following mechanisms: 1) rupture of the cystic artery due to sclerotic changes in the arterial wall, 2) mechanical irritation of the gallbladder wall due to the presence of a calculus, 3) interference of venous return at the gallbladder neck secondary to an impacted, 4) severe inflammation of the gallbladder mucosa with associated gangrene [3–5]. Long-term use of anticoagulants is also associated with increased risk of spontaneous rupture of the gallbladder [6]. Additional predisposing factors include postprandial gallbladder distention, a thin gallbladder wall, malposition of the gallbladder, and alcohol consumption [7].

In the present case, the patient suffered from an episode of abrupt and severe abdominal pain without any history of associated trauma, cholecystitis or biliary colic. Imaging was significant only for a small stone in the neck of the gallbladder, which could have caused acute cholecystitis and gallbladder gangrene with subsequent gallbladder rupture. However, there was no evidence of any acute inflammation of the gallbladder on the laparoscopic exploration. Moreover, the pathology also verified only the presence of chronic inflammation of the gallbladder mucosa.

Reviewing the patient’s past medical history, the patient had been taking aspirin 200 mg daily for three years after interventional treatment of a cerebral aneurysm. While there was no evidence of any coagulopathies on laboratory examination, aspirin use has been associated with complications including soft tissue edema, skin hemorrhage and hematuria. In the literature, the rupture and hemorrhage from the gallbladder is a rare complication, with only one previously-reported case of a gallbladder hematoma in a patient with hemophilia B. In this previous case, the diagnosis was made on MRI and a cholecystectomy was performed [8].

In the present case, the chronic inflammation of the gallbladder may have weakened the wall. Moreover, given the lack of collateral vascular supply to the gallbladder, even a small amount of bleeding may lead to gallbladder ischemia and potentially triggering the wall rupture. Furthermore, the calculus in the neck of the gallbladder could have also affected the blood supply of the gallbladder. In addition, the patient has long term use history of aspirin, which has been clarified clearly to prolong bleeding time and inhibit platelet aggregation [9]. Several reports that link coagulopathy and hemorrhagic cholecystitis have been published [10–12]. Thus, in this case, aspirin therapy is thought to be a predisposing factor for gallbladder hemorrhage, and continuous massive bleeding increase the pressure of gallbladder and even have exacerbated to rupture.

In conclusion, while the exact cause of the rupture of the gallbladder remains unclear in this case, the calculus in the gallbladder neck and the presence of chronic cholecystitis are significant risk factors for rupture. Long-term aspirin use may have further increased this risk. Gallbladder rupture and hemorrhage is a lethal condition, and rapid diagnosis and timely surgical intervention are the most important measures to treat the patient.

## Abbreviations

CT: Computed Tomography
